# Personality and Augmenting/Reducing (A/R) in auditory event-related potentials (ERPs) during emotional visual stimulation

**DOI:** 10.1038/srep41588

**Published:** 2017-02-06

**Authors:** Vilfredo De Pascalis, Francesca Fracasso, Philip J. Corr

**Affiliations:** 1 Department of Psychology, La Sapienza University of Rome, Italy; 2 Department of Psychology, City, University of London, United Kingdom

## Abstract

An auditory augmenting/reducing ERP paradigm recorded for 5 intensity tones with emotional visual stimulation was used, for the first time, to test predictions derived from the revised Reinforcement Sensitivity Theory (rRST) of personality with respect to two major factors: behavioral inhibition system (BIS), fight/flight/freeze system (FFFS). Higher BIS and FFFS scores were negatively correlated with N1/P2 slopes at central sites (C3, Cz, C4). Conditional process analysis revealed that the BIS was a mediator of the association between the N1/P2 slope and the FFFS scores. An analysis of covariance showed that lower BIS scorers exhibited larger N1/P2 amplitudes across all tone intensities while watching negative, positive and neutral pictures. Additionally, lower FFFS scorers compared to higher FFFS scorers disclosed larger N1/P2 amplitudes to the highest tone intensities and these differences were even more pronounced while watching positive emotional pictures. Findings were explained assuming the operation of two different, but related processes: transmarginal inhibition for the BIS; the attention/emotional gating mechanism regulating cortical sensory input for the FFFS trait. These findings appear consistent with predictions derived from the rRST, which traced fear and anxiety to separate but interacting neurobehavioural systems.

One of the most known neuroscience theories of personality is the reinforcement sensitivity theory (RST)^1^,^2^,^3^,^4^. The original formulation of the RST^5^ set emphasis upon only two neurobehavioural systems, the behavioural inhibition system (BIS) and the behavioural approach system (BAS). There is a hidden complexity in and between these systems^6^ and this is captured in the revised RST (rRST) which postulates three major neuropsychological systems, two for defensive behaviours (the *fight-flight-freeze system*, FFFS; and the *behavioural inhibition system*, BIS), and one for approach behaviours (the *behavioural approach system*, BAS)^7^. The BAS is activated by all and any appetitive stimuli; FFFS is activated by all and any aversive stimuli; and the BIS is activated by stimuli indicating conflict between goals (e.g., co-activation of FFFS and BAS, or whether to take flight or freeze to avoid a punishing stimulus). The most significant change in rRST is the separation of FFFS-fear and BIS-anxiety processes, which are postulated to have different functional properties and distinct neuropsychopharmacological bases^1^,^3^,^4^. McNaughton and Corr^3^ proposed that the ‘direction’ of defensive behaviour could be taken to distinguish FFFS from BIS: the FFFS is active when threat needs only to be avoided (defensive avoidance), or escaped from, while the BIS is active when a threatening situation requires defensive approach. Although these two systems were contained in the early version of RST^5^, they were not adequately distinguished or defined. In line with this two-dimensional view, different types of evidence support FFFS/BIS separability. Predictive validity studies of fear and anxiety point to their different functions in human behaviour^8^. Psychopathological research has highlighted the need of FFFS/BIS separation^9^, where the absence of useful psychometric measures of fear and anxiety has been outlined as a significant hindrance to research advancement^10^. Very recently, consistent with both theoretical and empirical considerations of the rRST, a new questionnaire has been proposed, the Reinforcement Sensitivity Theory of Personality Questionnaire (RST-PQ)^11^, developed on the basis of qualitative responses to defensive and approach scenarios. The RST-PQ revealed a robust 6-factor structure: 2 unitary defensive factors, the FFFS, related to fear, and the BIS, related to anxiety; and four BAS facets (Reward Interest, Goal-Drive Persistence, Reward Reactivity, and Impulsivity).

The aim of this study was to examine, for the first time, how ERPs elicited using an auditory augmenting/reducing (A/R) paradigm^12^ with emotional visual stimulation can be used to differentiate the defensive systems in terms of BIS and FFFS.

The A/R is a measure of sensory inhibition and is assumed to reflect individual differences in the modulation of sensory input. Buchsbaum and Silverman^12^ developed a visual ERP paradigm using flashes at different levels of stimulus intensity, and ERP amplitudes were correlated with the logarithm of flash intensity. Research has demonstrated that A/R phenomenon at high intensities of stimulation reflects cortical inhibition^13^. This is essential for the filtering properties of a gating mechanism that regulates sensory input to the cerebral cortex^12^,^14^. Individuals are typically classified as augmenters or reducers according to whether they show an increase or decrease on evoked potential amplitudes with increasing of stimulus intensity. The amplitude-stimulus function (ASF) has been suggested as index of individual modes of processing sensory input^15^. The ASF is defined as the slope of the linear regression line for the individual P1, N1, P2, P1/N1 and N1/P2 amplitudes across the 5–6 stimulus intensities^16^,^17^,^18^. Peak measures of the auditory ERP components are considered reliable measures for the assessment of A/R, both in terms of internal consistency and temporal stability^19^,^20^,^21^. One of the replicated auditory A/R findings in this area is a positive correlation between personality trait of sensation seeking (SS) and ASF slope. In addition, consistently with Eysenck’s theory of extraversion and arousal^22^, augmenting has been associated with the personality traits of extraversion and, conversely, reducing with introversion^23^. Consistent with these findings, augmenters of the ERPs tend to be high sensation seekers, and reducers tend to be sensation avoiders^13^,^16^,^17^,^24^,^25^,^26^,^27^. Augmenting of the auditory N1/P2 amplitude has been suggested to reflect low central serotonergic neurotransmission, and vice versa^15^, a finding which appears in line with observations that responders to antidepressant or prophylactic medication are augmenters, whereas non-responders tend to be reducers^14^,^15^,^28^,^29^.

While A/R research has been focused almost exclusively on individual differences in extraversion and sensation-seeking, to our knowledge there are no studies linking BIS/FFFS traits to A/R of the ERPs during positive and negative emotional processing. In terms of emotional processing in humans, Lang^30^ suggests an emotional system subdivided into aversive and appetitive motivational systems. The former facilitates defensive behaviour, such as avoidance, escape or defence, whereas the latter facilitates approaching behaviours, such as mating, food taking or exploration. Lang and colleagues^31^ conceptualize valence and arousal as the fundamental dimensions of the emotions: valence determines the direction, and arousal the intensity of activation. A main aim of the present study was to account for the possible effect of the interaction between affective image content and individual differences in defensive BIS and FFFS functioning on the A/R of the ERPs – this we achieved by using the RST-PQ^11^ that allows the separation of the FFFS and BIS. It is important to establish these relations because RST has largely superseded Eysenck’s arousal theory of personality and incorporated Zuckerman’s SS factor under BAS Impulsivity.

In the rRST^1^, the BIS is a system that not only amplifies attention, but also arousal^5^ as well as amplifying inhibition of behaviour. On this basis, we used a visual cue indicating, 2 sec in advance, whether participants would see an emotional positive, negative or neutral picture. We expected that high BIS individuals, being more sensitive to negative stimuli, they should be more likely to reduce their activation, especially to afford the activation induced by the negative pictures, while individuals with low BIS should be more prone to enhance ERP responses to the highest auditory intensities while watching positive pictures. We expect much less of an influence of the FFFS as there is no immediate danger, and the stimuli are likely to engage more complex motivational and emotional processes with some degree of goal-conflict attached. Thus, we expected a significant negative correlation between N1/P2 amplitude and BIS scores. This association should be apparent, although less consistent, also for FFFS scores. We expected smaller enhancements in N1 and P2 or N1/P2 amplitudes with increasing stimulus intensity in high BIS, as compared low BIS individuals. These differences should be more pronounced for participants exposed to unpleasant pictures^32^,^33^. Given the high reliability of N1/P2 slope^34^ we also expected a significant negative association of this measure with BIS and, although less consistent, with FFFS measure.

The BIS and FFFS are likely to interact^7^. Risk factors, labeled ‘Distress’ and ‘Fear’, assumed to reflect BIS and FFFS sensitivities^35^, are found strongly correlated although appear to have a distinct genetic basis^36^,^37^. Moreover, RST theory predicts that highly approach-oriented (i.e., high BAS) individuals should be augmenters of N1 and P2 waves of the ERPs in response to increased levels of auditory stimulus intensity. This hypothesis is supported by a number of personality findings showing that high scores on action-oriented personality traits, such as sensation seeking, extraversion, and impulsivity, are associated with higher ASF of the auditory and visual ERPs^19^,^26^,^38^. Although, ERP correlates of BAS and its facets are reported in another study^39^, here we enclosed a measure of sensation seeking^40^ to report our ERP-A/R correlates of sensation seeking.

## Results

### Affective Ratings

Valence and arousal ratings are reported in Table 1. The ANOVA performed on the valence ratings yielded a main effect of valence: F2,76 = 1076.29, p < 0.0001, η^2^ = 0.84. As expected, negative images and positive images were rated as more unpleasant and pleasant, respectively, than were neutral images (see Table 1).

The ANOVA performed on the arousal ratings revealed the expected interaction between valence and arousal, F2,76 = 9.71, *p* < 0.0001, η^2^ = 0.83. Negative images (M = 6.20, SD = 0.77) and positive images (M = 6.12, *SD* = 0.76) were rated as more arousing than neutral images (M = 4.21, SD = 0.69), *p* < 0.0001, whereas negative and positive images were evaluated as equally arousing (p > 0.05). Separate ANCOVA analyses on affect and arousal ratings, using each RST trait of interest as a covariate, failed to reveal significant effects involving the RST traits, were all p_s_ > 0.05 (Table 1). The correlation matrix of the RST measures of interest and SS scores with emotional and arousal ratings are available as Supplementary Information (section S1).

### N1/P2 complex

**Figure 1:**
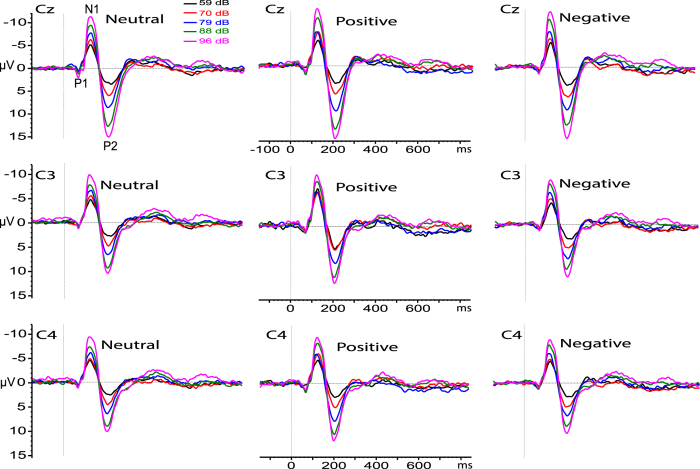
Grand average ERPs obtained on 39 participants at recording sites C3, Cz, and C4, for five different tone intensities delivered during the presentation of neutral, positive and negative pictures. The ERP components P1, N1 and P2 are indicated for auditory intensities of 59, 70, 79, 88, 96 dB SPL.

The valence and arousal ratings are reported in Table 1. The ANOVA preformed on N1/P2 amplitude scores found a main effect for valence, F2,76 = 4.53, p = 0.014, η^2^ = 0.14, indicating a larger N1/P2 amplitude for positive compared to neutral and negative valences, 18.6 vs. 17.6 and 17.5 μV, respectively. The main effects of stimulus intensity, recording site, and their interactions were all highly significant, F4,152 = 96.29, p < 0.0001, η^2^ = 0.82; F2,76 = 68.75, p < 0.0001, η^2^ = 0.73; and F8,304 = 5.06, p < 0.0001, η^2^ = 0.72. These effects indicate that N1/P2 amplitude increased the function of stimulus intensity, with the largest increase occurring at 88 and 96 dB at Cz recordings. The interaction of valence with recording site was significant, F4,152 = 2.68, p = 0.034, η^2^ = 0.23, indicating that for positive valences, the Cz site exhibited a larger N1/P2 amplitude compared to negative and neutral pictures (Fig. 1).

### Personality measures

Pearson correlation coefficients among personality and state anxiety measures are reported in Table 2. The BIS measure, as expected, was significantly associated with the FFFS and state anxiety scores^11^. SS, as measured by the ZKAPQ, was not significantly associated with any of the RST-PQ measures, although the negative association with the FFFS and the positive association with BAS-TOT approached the level of significance.

### Relationship between N1/P2 amplitudes and RST traits

We obtained significant zero-order correlations between RST traits and N1/P2 amplitude only for the highest intensity tone (96 dB). For this auditory intensity, the BIS was significantly correlated with the N1/P2 amplitude during the presentation of positive pictures, respectively, at C4, C3, and Cz [C4 lead: r = −0.50 (95%CI = −0.62, −0.30); C3 lead: r = −0.49 (95%CI = −0.61, −0.31); and Cz lead: r = −0.45 (95%CI = −0.58, −0.26), p < 0.01]. With respect to the negative pictures, this association was significant at the C4 lead [r = −0.45 (95%CI = −0.57, −0.26)], whereas during the presentation of the neutral pictures, this association was significant at both the C3 and C4 leads [C3: r = −0.49 (95%CI = −0.61, −0.29); C4: r = −0.52 (95%CI = −0.55, −0.22), p < 0.01]. The FFFS scores were significantly associated with the N1/P2 amplitude of C4 at 96 dB tone during the presentation of positive pictures [r = −0.41 (95%CI = −0.54, −0.22)].

No significant correlations were found between ZKAPQ-SS and N1/P2 amplitudes (r values ranged from −0.07 to −0.22).

### Correlations between RST traits and P1/N1 and N1/P2 slopes

The correlations of P1/N1 and N1/P2 slopes (across neutral, positive, negative pictures, as well as the overall averaged measure) with the RST traits of interest and their 95% associated bootstrapped confidence intervals are reported in Table 3. The P1/N1 slope was significantly and negatively correlated with the BIS measures but not with the FFFS measure. The N1/P2 slope also yielded significant and negative correlations with the BIS and FFFS scores (Table 3).

### ASF correlates of FFFS using BIS as a moderator and mediator variable

Because the highest correlation coefficients of FFFS occurred with the N1/P2 slope at C4 during positive pictures (Table 3), we tested the role of the BIS as a moderator and mediator of the association of the ASF measure with the FFFS scores used as criteria. We tested these effects by using the conditional process analysis^41^. The moderating effect of the BIS was not significant (p > 0.05), but the process analysis indicated that the BIS was a significant mediator of the above-mentioned relationship. In particular, we obtained significant direct effects of the N1/P2 slope on the FFFS outcome (B = −15.54, se = 4.96, t = −3.14, p = 0.0034) and on the BIS outcome (B = −36.31, se = 1.97, t = −3.31, p = 0.0021). The direct effect of the BIS in predicting the FFFS outcome was significant (B = 0.24, se = 0.06, t = 3.77, p = 0.0006), and finally, the indirect effect of the BIS as a mediator of the N1/P2 slope vs. FFFS outcome was also significant (indirect effect = −8.73, se = 3.58, z = −2.44, p = 0.0147).

### RST traits and N1/P2 complex

**Figure 2:**
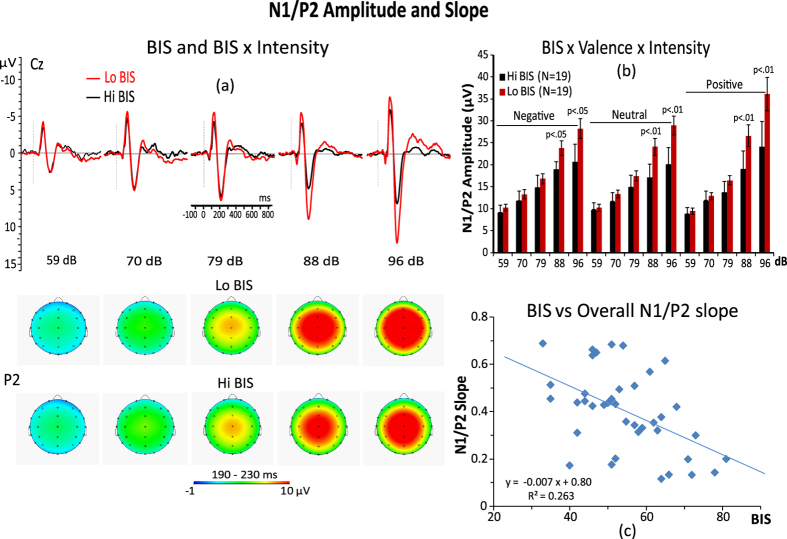
(**a**) Grand average midline ERP responses and scalp maps of P2 amplitude for 5 tone intensities (59, 70, 79, 88, 96 dB SPL) in high and low BIS participants (left panel). (**b**) Histogram in the upper-right panel is displaying the interaction of BIS, Valence of the picture, and Intensity of the tone on overall N1/P2 amplitude. (**c**) Scatterplot in the bottom-right panel depicts the relationship between BIS and overall N1/P2 slope.

The ANCOVA performed on the N1/P2 peak amplitude, entering the BIS trait as a covariate and valence (positive, negative, neutral) by electrode site^17^ (C3, Cz, C4) by stimulus intensity (59, 70, 79, 88, and 96 dB-SPL) as within-subject factors, disclosed smaller N1/P2 amplitudes in high-BIS participants compared to low-BIS participants, F1,37 = 6.82, p = 0.013, η^2^ = 0.18. The interaction of the BIS with stimulus intensity was also significant, F4,148 = 9.90, p < 0.001, η^2^ = 0.312. Follow-up contrasts indicated that the auditory intensity of 88 and 96 dB elicited a significantly smaller augmenting effect of the N1/P2 amplitude in high-BIS participants compared to low-BIS participants (p < 0.01). These effects are displayed in Fig. 2. Further, the interaction of the BIS with valence, F2,78 = 4.45, p = 0.028, η^2^ = 0.18, was significant. Moreover, the interaction of these two factors with stimulus intensity, F4,148 = 3.19, p = 0.028, η^2^ = 0.23, was also determined to be significant (Fig. 2a). This interaction revealed that during both negative and neutral pictures, low-BIS participants exhibited significantly larger N1/P2 amplitudes than did high-BIS participants for the 96 dB tone, whereas, with respect to positive pictures, differences between BIS groups were found for both the 88 and, even more pronounced, for the 96 dB tones (Fig. 2b).

A similar ANCOVA performed on the N1/P2 slope scores found a highly significant main effect for the BIS, F1,37 = 13.12, p = 0.0009, η^2^ = 0.35, indicating lower slopes for high-BIS individuals compared to low-BIS participants (Fig. 2c).

**Figure 3:**
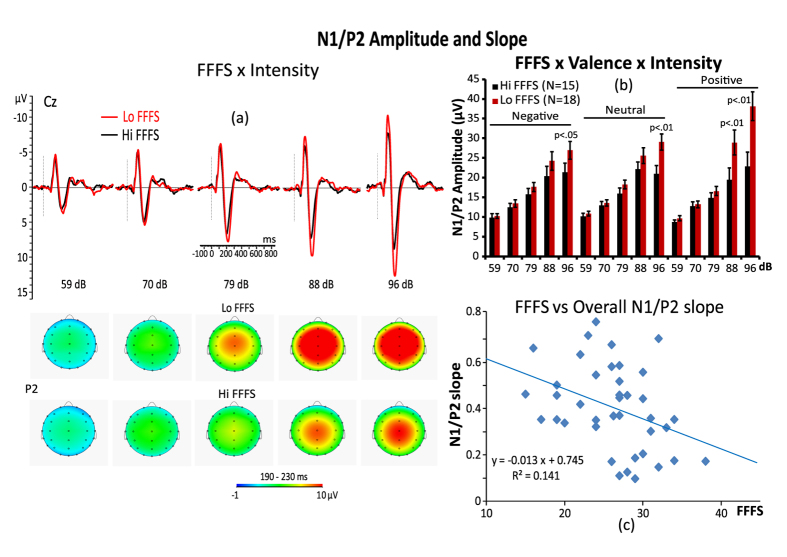
(**a**) Grand average midline ERP responses and scalp maps of P2 amplitude for 5 tone intensities (59, 70, 79, 88, 96 dB SPL) in high and low FFFS participants (left panel). (**b**) Histogram in the upper-right panel is displaying the interaction of FFFS, Valence of the picture, and Intensity of the tone on overall N1/P2 amplitude. (**c**) Scatterplot in the bottom-right panel shows the relationship between FFFS and overall N1/P2 slope.

The ANCOVA using the FFFS scores as a covariate showed a significant interaction of the FFFS with stimulus intensity, F4,148 = 6.89, p = 0.006, η^2^ = 0.17. Follow-up contrasts indicated that for the 96 dB tones, there was a significantly smaller N1/P2 amplitude enhancement in the high-FFFS participants compared to the low-FFFS participants (p < 0.01). This effect is displayed in Fig. 3a. The second-order effect of the FFFS with valence and the third-order interaction of the FFFS with stimulus intensity and valence were both significant, F2,74 = 3.41, p = 0.038, η^2^ = 0.11 and F4,148 = 3.71, p = 0.014, η^2^ = 0.18, respectively. A contrast analysis revealed that low-FFFS participants, compared to high-FFFS participants, had significantly larger N1/P2 amplitudes for the 96 dB tones during the presentation of negative pictures and for both the 88 and 96 dB tones during both neutral and positive pictures, although the larger differences were found for positive pictures (Fig. 3b).

The analysis of the N1/P2 slope data found a highly significant main effect for the FFFS, F1,37 = 6.50, p = 0.015, η^2^ = 0.17, indicating lower slopes in high-FFFS participants compared to low-FFFS participants (Fig. 3c).

LORETA source localisation findings on individual differences of BIS and FFFS traits are available in the Supplementary Information, section S2.

## Discussion

The findings of this study corroborate the view that auditory A/R, as traditionally defined, is related to the temperamental traits of the BIS and the FFFS, as measured by the RST-PQ. Correlation analyses showed clearly that a higher BIS was uniquely and negatively related to the N1/P2 slope and that high-BIS participants had smaller increases in N1/P2 amplitudes than did low-BIS participants. These findings also extend original clinical reports that patients with unipolar affective disorders often showed decreases in ERP amplitude with increasing stimulus intensity^14^,^17^.

Unfortunately, the classical finding regarding the significant association of N1/P2-ASF slope and SS was not replicated. Because the positive correlation between augmenting and SS has been extensively replicated using the SS Scale Form V (SSS-V)^16^,^17^ for both genders, we are compelled to conclude that the SS measure of the ZKA-PQ used in the present study may account for our lack of findings.

With respect to the N1/P2 amplitudes, we observed that participants with lower BIS and FFFS scores had larger amplitudes to the highest tone intensities during the presentation of negative, positive and neutral pictures, although these differences were more pronounced in lower FFFS scorers compared to higher FFFS scorers while watching positive emotional pictures at the highest tone intensities (Figs 2b and 3b). These findings, in the whole, indicate that, at least at the processing levels expressed by the N1/P2 complex, the BIS and FFFS are similarly sensitive to stimulus intensity and emotional valence processing and that, both positive emotional valence and stimulus intensity had an additive effect on N1/P2 amplitude enhancement in lower FFFS scorers. But, the most pronounced positive emotion effect on N1/P2 amplitude, found for the FFFS factor, indicates that BIS and FFFS are not similarly sensitive to positive emotions. Thus we think that two different, but related processes, are responsible for stimulus intensity and emotional differences between BIS and FFFS factors. Furthermore, the larger N1/P2 complex found in low BIS and, for the highest tone intensities, also in low FFFS appears to contradict Gray’s (1982) theory that a higher BIS and FFFS should be associated with larger ERP responses to negative emotions. Mainly they seem to conflict with previous findings of a startle response in high harm-avoidance scorers^42^,^43^ and with fear images in high-BIS participants^44^. We think that these apparent contrasting findings can be explained if we assume that a regulatory mechanism of the sensory input was at work in high BIS participants, while they were watching negative pictures, analogous to the Pavlovian mechanism of transmarginal inhibition of response, which is a protective process that operates at high levels of sensory stimulation^12^. This view in line with the conceptualization that higher-BIS individuals are devoted to activate the scanning of memory and environment to help resolve concurrent conflict. At high stimulus intensities, protective inhibition would act, in concordance with passive avoidance behavior, to avert the threat of sensory overload by dampening the impact of the sensory and emotional input. On the other side, rather than transmarginal inhibition, the process accounting for individual differences in FFFS should involve the “tuning” properties of an attention/emotional gating mechanism that regulates cortical sensory input^45^,^46^,^47^. More specifically, higher FFFS participants were more prone to reduce N1/P2 amplitude by a selective attention/emotional mechanism acting as an inhibitory gate controlling ascent of sensory information to the cortex in function of the auditory stimulus intensity and unpleasantness of negative picture^48^. This view is in line with a number of A/R studies suggesting a selective activation of the thalamic reticular formation by the prefrontal-mediated inhibition of sensory processes^46^,^49^,^50^. A short discussion of LORETA findings on individual differences of BIS and FFFS traits is also provided in the Supplementary Information, section S3.

As expected, we have found the BIS highly correlated with FFFS (0.63, Table 2). Yet, we have proved that BIS is a significant mediator of the association of N1/P2 slope with FFFS. We think that this finding demonstrates the validity of McNaughton and Gray^7^ view that conceptualized the interaction between BIS and FFFS. These findings parallel clinical reports of a robust association between distress (expression of the BIS) and fear, although they have a distinct genetic basis^36^. These findings are also aligned with our prior auditory startle observations showing higher fear levels associated with smaller P2 amplitudes, with anxiety influencing this relation^51^.

The present findings can be considered as an extension to a normative sample of previous clinical reports of reduced P2/P3 amplitudes^52^ and EEG-cortical excitability^53^ in PTSD patients. On the whole, our findings indicate that BIS and FFFS have different underlying electrocortical mechanisms and appear in line with predictions derived from the revised RST^2^ that trace fear and anxiety to separate but interacting brain systems.

A limitation of the present study lies in the fact that our findings are restricted to women participants and, thus, cannot be generalized to men. Further studies are necessary to replicate the present findings by considering gender and state emotionality measures as potential variables influencing the association between RST traits, and ERP responses.

In conclusion, the present findings indicate that these BIS and FFFS traits involve separate but interacting neurophysiological systems that together allow the individual to avoid threats, as is necessary for the sustenance of life^3^.

## Methods

### Participants

Forty healthy, right-handed women graduate students participated in the study (19 to 33 years; mean age = 24.76, SD = 3.0). The sample was restricted to female students to avoid possible gender differences as a confounding factor in augmenting/reducing the ERPs. Inclusion criteria required the absence of any lifetime history of hearing problems and no history of neurological illness or drug abuse. To avoid a possible effect of the menstrual cycle on auditory ERPs, participants who were in a menstrual period were asked to report for their EEG recordings between the 5th and 11th day after the onset of menses. All participants were strictly right-handed as assessed by the Edinburgh Inventory and were asked to refrain from smoking or drinking coffee for at least three hours before the EEG recording. The experiment was conducted according to the Declaration of Helsinki (BMJ 1991; 302: 1194) after approval from the Ethical Committee of the Department of Psychology, La Sapienza University of Rome, and in compliance with APA ethical standards for the treatment of human volunteers (1992, American Psychological Association). All participants gave written informed consent prior to their participation in the experiment.

### Questionnaires

The study used the RST-PQ^11^, a recently developed instrument consisting of 71 statements that measure three major systems conceptualised in the revised RST: fight/flight/fear system (FFFS); behavioural inhibition system (BIS); and behavioural approach system (BAS). Participants were asked how accurately each statement described them based on a scale from 1 (not at all) to 5 (highly).

Cronbach’s α values for the BIS and the FFFS subscales were. 90 and 78, respectively.

The State Anxiety Inventory (STAI-Y1^54^) was used to assess state anxiety by presenting 20 items that asked respondents indicate how they felt in the moment about a given situation. The scale ranged from 1 (not at all) to 4 (very much so).

In addition, SS was measured using the Zuckerman–Kuhlman–Aluja Personality Questionnaire^40^ (α = 0.74).

### Emotional stimuli

The images were selected from the International Affective Picture System (IAPS^55^). For the selection, we invited 30 female psychology students (22 to 36 years, M = 24.6, SD = 2.6 years, N = 30) to rate each image on valence and arousal. Details of the picture selection procedure and IAPS picture numbers are available in the Supplementary Information (section S4). The ratings for positive, negative and neutral valences were obtained using a 9-point Likert scale that ranged from 1 (negative) to 9 (positive) with a neutral point of 5. A similar scale that ranged from 1 (calm) to 9 (arousing) was used to rate arousal levels. We selected from the IAPS all the positive and negative pictures that had a score equal to or higher than 7 in valence and 7 in arousal. Because neutral images are typically rated lower in arousal relative to positive or negative images, the maximum values we were able to obtain for the neutral images were in an interval of approximately 5 for the valence (4.5 to 5.5) and approximately 4.5 for arousal (4.3 to 5.5). These selected images were then administered to the present experimental sample. Emotional valence and arousal of the experimental sample (N = 39) for positive, negative and neutral images are reported in Table 1. These ratings correspond to the ratings for women reported in the validation study by Bradeley and Lang^56^. More statistical details for valence and arousal ratings are available in the Supplementary Table S1.

### Acoustic stimuli and trial structure

The acoustic stimuli were delivered binaurally through headphones (Telephonics) by using STIM^2^ (NeuroScan Inc., Herndon, VA, USA) during the presentation of emotional and neutral images. Acoustic stimuli consisted of a pseudo-randomised presentation of 1000 Hz tones at five different stimulus intensities (59, 70, 79, 88, and 96 dB-SPL). Each auditory stimulus lasted 30 ms (10 ms rise and 10 ms fall time). The interstimulus interval (ISI) varied pseudo-randomly between 1600 and 2100 ms.

Before the electrophysiological recording began, all participants were screened for intact auditory abilities. Participants had to be excluded on the basis of hearing impairment at 40 dB(A) (1000 Hz). All participants passed this screening. They were then comfortably seated in an armchair placed in a sound attenuated room near the recording equipment. After an initial 5 min recording of resting EEG, five auditory tones were delivered during the presentation of each picture according to the A/R paradigm^12^.

**Figure 4:**
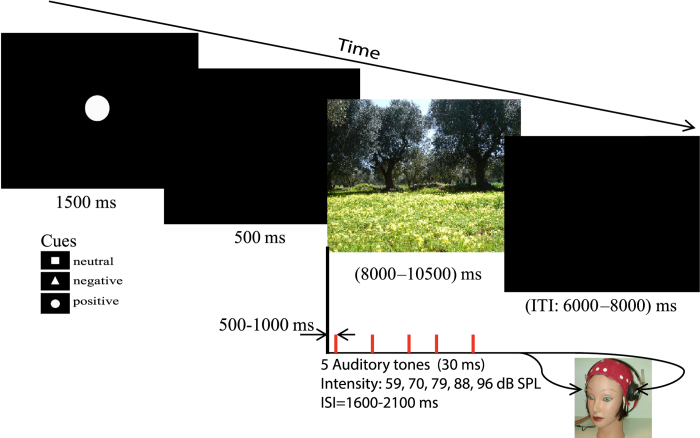
Schematic diagram illustrating visual emotional display, timing, and auditory stimulation using the augmenting/reducing paradigm.

A schematic view and the time course of a trial are shown in Fig. 4. All stimuli were viewed at a visual angle of 7.5° × 7.5° and were presented on a monitor with a frame rate of 75 Hz (luminance of approximately 200 cd/m^2^). Each trial began with a 1500 ms presentation appearing in the centre of the computer screen. The presentation was of one of the following three fixation cue stimuli: a white dot circle (2 cm diameter), an equilateral triangle (3 cm side), or a square (2 cm side), and the cue indicated that a positive, negative, or neutral image, respectively, would be displayed. A blank screen then appeared for 500 ms, and an emotional image was next presented on the screen for a time period ranging, in pseudorandom order, from 8 to 1.5 seconds to guarantee the presentation of five different intensity tones. Each picture presentation was followed by an intertrial interval (ITI) varying between 6 to 8 seconds (blank screen). The first of five tone probes occurred 500 ms after the onset of each image presentation. The duration of each trial varied between 1.6 and 2.5 seconds.

The images were presented in pseudo-random order in five blocks with a 1-min rest between blocks. An equal number of images from each category occurred in each block. The duration of each block was approximately 10 minutes, and 30 pictures were presented in each block.

### EEG Recordings and analysis

The EEG and electro-ocular (EOG) data were acquired simultaneously and continuously, using a 40-channel NuAmps DC amplifier system (Neuroscan Acquire 4.3) with tin electrodes located over 30 scalp sites (Fp1, Fp2, F7, F3, Fz, F4, F8, FT7, FC3, FCz, FC4, FT8, T3, C3, Cz, T4, C4, T5, CP3, CPz, CP4, T6, P3, Pz, P4, TP7, TP8, O1, Oz, O2) and grounded using a midline electrode positioned 10 mm anteriorly to the Fz lead. Linked earlobes [(A1 + A2)/2] were used as a reference electrode. Amplifiers were set at a gain of 200, a sampling rate of 1000 Hz, and with signals band-limited from 1 to 48 Hz (Butterworth zero phase filter, 24 dB/octave roll off). Electrode impedance was kept below 5 kΩ. The horizontal and vertical EOG was monitored via a pair of tin electrodes placed 1 cm lateral to the outer cantus of each eye, and the vertical EOG was monitored via a bipolar montage using two electrodes placed above and below the centre of the left eye. The EEG was offline processed using the Brain Vision Analyzer 2.1 system (Brain Product). The EEG was reconstructed into discrete, single-trial 1000-ms epochs, and the ERPs were time-locked to auditory tone onset with a 150-ms pre-stimulus baseline. Trials that contained eye blink or eye movement artefacts (EOG > 75 μV) were rejected and discarded from analysis. Ocular artefacts were corrected using the procedure of Gratton and colleagues^57^. To ensure an acceptable signal-to-noise ratio in the averaged ERP waveforms, only subject data including no less than 50 artefact-free epochs per condition were included. Based on this criterion, one individual was dropped from the initial 40. Thus, 39 participants were included in all the analyses.

There were no differences between affective conditions in the number of rejected trials. The EEG was averaged for each stimulus intensity and affective condition and then baseline corrected. Peak amplitudes were determined for the P1 as the most positive peak within the period of 30–80 ms (M = 65.8, SD = 5.2 ms), for the N1 as the most negative peak within 80–140 ms (M = 126.9, SD = 9.9 ms), and for P2 as the most positive peak within 140–250 ms (M = 211.4, SD = 11.5 ms). Additional peak-to-peak values were calculated for P1/N1 and N1/P2.

ERP responses were further analysed using the last LORETA software to compute statistical maps using EEG data that indicate the locations of the putative underlying source generators^57^,^58^. Details of the Loreta method are available as Supplementary Information (sections S5).

Zero-order correlations were obtained to evaluate the relation between the P1/N1, N1/P2 amplitudes and the P1/N1 and N1/P2 slopes with the BIS and FFFS traits. The significance of these correlations was assessed by using the bias-corrected bootstrap method, which is effective in controlling for type 1 errors associated with multiple comparisons^58^. This bootstrap analysis estimates critical values for the upper and lower 95% bias-corrected confidence limits for all the zero-order correlation coefficients. All coefficients with an associated confidence interval that did not include zero and were above the level of significance (α = 0.01) were considered statistically significant.

We also tested the role of the BIS as a moderator or mediator of the ASF measure used as predictor of the FFFS scores. We tested this effect by using the conditional process analysis^41^. Because adding the interaction term to the equation may introduce considerable multicollinearity, which may lead to problems estimating the regression coefficients, the continuous predictors were centred, i.e., a mean of zero was computed by subtracting the mean of the variable from each value^59^. The PROCESS macro (www.afhayes.com) was used in all regression analyses.

To test for differences in the self-report emotional valence, an analysis of variance (ANOVA) was performed with valence (positive, negative, neutral) as within-subject factors. A similar ANOVA was used for arousal levels. To examine the influence of the RST personality traits on the P1/N1 and N1/P2 amplitude measures, separate repeated measures ANCOVAs were computed by using each trait as a covariate and valence (positive, negative, neutral) by electrode site^17^ (C3, Cz, C4) by stimulus intensity (59, 70, 79, 88, and 96 dB-SPL) as within-subject factors. The P1/N1 and N1/P2 slopes were subjected to similar separate ANCOVAs. An alpha level of 0.05 was used for all analyses. Bonferroni corrected follow-up comparisons were conducted to assess effects of picture type and electrode location.

## Additional Information

**How to cite this article**: De Pascalis, V. *et al*. Personality and Augmenting/Reducing (A/R) in auditory event-related potentials (ERPs) during emotional visual stimulation. *Sci. Rep.* **7**, 41588; doi: 10.1038/srep41588 (2017).

**Publisher's note:** Springer Nature remains neutral with regard to jurisdictional claims in published maps and institutional affiliations.

## Supplementary Material

Supplementary Information

## Figures and Tables

**Table 1:** Mean (M) and Standard Deviation (SD) of Emotional Valence and Arousal ratings.

Variable	M	SD
**Negative Valence**	2.44	0.49
**Positive Valence**	6.91	0.37
**Neutral Valence**	5.29	0.45
**Negative Arousal**	6.20	0.77
**Positive Arousal**	6.12	0.76
**Neutral Arousal**	4.21	0.70

**Table 2:** Pearson correlation coefficients and descriptive statistics for personality and state anxiety (N = 39).

	BIS	FFFS	ZKAPQ-SS	BAS-TOT	STAI-Y1
**BIS**	1				
**FFFS**	0.63† (0.53, 0.75)	1			
**ZKAPQ-SS**	−0.02 (−0.15, 0.19)	−0.34 (−0.53, 0.003)	1		
**BAS-TOT**	0.12 (−0.09, 0.38)	0.09 (−0.10, 0.35)	0.31 (0.11, 0.37)	1	
**STAI-Y1**	0.47* (0.33, 0.66)	0.16 (−0.03, 0.44)	0.11 (−0.10, 0.35)	−0.15 (−0.38, 0.10)	1
Mean	54.8	26.2	98.8	91.3	37.7
SD	11.8	5.3	11.2	9.6	7.9
Range	33–81	15–38	69–134	72–110	25–57

Bootstrapped 95% confidence interval is reported in parentheses.

*Note-* Personality Measures: Reinforcement Sensitivity Theory Personality Questionnaire. (RST-PQ; Corr and Cooper, 2015); STAI-Y1: state anxiety (Spielberger *et al*.^54^); ZKAPQ-SS: Sensation Seeking (Aluja *et al*.^40^). *p < .01; ^†^p < .0001.

**Table 3:** Zero-order correlations, along with their 95% associated bootstrapped confidence intervals (CI), of the P1/N1 and N1/P2 slopes for Neutral (NEU), Negative (NEG), Positive (POS) pictures, and Overall Averaging (Avg) with BIS and FFFS measures of the RST-PQ and SS subscale of the ZKAPQ questionnaire.

	BIS		FFFS		ZKAPQ-SS	
**P1/N1 slope**	r	95%CI	r	95%CI	r	95%CI
** C3_NEU**	−0.27	(−0.45, −0.01)	−0.02	(−0.20, −0.24)	−0.19	(−0.38, 0.05)
** Cz_NEU**	−0.38	(−0.53, −0.14)	−0.05	(−0.21, 0.17)	−0.23	(−0.38, 0.04)
** C4_NEU**	−0.45*	(−0.58, −0.26)	−0.14	(−0.30, 0.09)	−0.17	(−0.32, 0.01)
** C3_NEG**	−0.41*	(−0.55, −0.18)	−0.18	(−0.33, 0.09)	−0.24	(−0.40, 0.01)
** Cz_NEG**	−0.37	(−0.52, −0.14)	−0.12	(−0.27, 0.11)	−0.25	(−0.39, 0.00)
** C4_NEG**	−0.43*	(−0.57, −0.18)	−0.19	(−0.35, 0.08)	−0.25	(−0.42, −0.07)
** C3_POS**	−0.45*	(−0.56, −0.27)	−0.26	(−0.42, −0.02)	−0.20	(−0.33, −0.03)
** Cz_POS**	−0.43*	(−0.57, −0.22)	−0.25	(−0.45, −0.02)	−0.26	(−0.40, −0.09)
** C4_POS**	−0.47*	(−0.58, −0.32)	−0.19	(−0.36, 0.00)	−0.33	(−0.47, 0.01)
** NEU Avg**	−0.38	(−0.53, −0.15)	−0.07	(−0.23, 0.17)	−0.21	(−0.45, −0.14)
** NEG Avg**	−0.42*	(−0.56, −0.18)	−0.16	(−0.32, 0.09)	−0.26	(−0.42, −0.09)
** POS Avg**	−0.52•	(−0.65, −0.33)	−0.27	(−0.44, −0.02)	−0.31	(−0.45, 0.02)
** Averall Avg**	−0.47*	(−0.61, −0.27)	−0.18	(−0.34, 0.08)	−0.25	(−0.41, −0.05)
**N1/P2 slope**						
** C3_NEU**	−0.47*	(−0.60, −0.24)	−0.33	(−0.46, −0.11)	−0.173	(−0.33, 0.02)
** Cz_NEU**	−0.49*	(−0.63, −0.27)	−0.26	(−0.41, −0.05)	−0.164	(−0.29, 0.01)
** C4_NEU**	−0.47*	(−0.61, −0.26)	−0.40	(−0.52, −0.20)	−0.057	(−0.20, 0.11)
** C3_NEG**	−0.39	(−0.55, −0.16)	−0.30	(−0.44, −0.08)	−0.146	(−0.28, 0.01)
** Cz_NEG**	−0.45*	(−0.59, −0.23)	−0.25	(−0.39, −0.05)	−0.161	(−0.32, 0.04)
** C4_NEG**	−0.46*	(−0.59, −0.25)	−0.43*	(−0.56, −0.21)	−0.026	(−0.18, 0.14)
** C3_POS**	−0.52•	(−0.64, −0.30)	−0.42*	(−0.57, −0.20)	−0.167	(−0.33, 0.03)
** Cz_POS**	−0.44*	(−0.59, −0.23)	−0.35	(−0.54, −0.14)	−0.209	(−0.35, 0.01)
** C4_POS**	−0.48*	(−0.62, −0.27)	−0.46*	(−0.58, −0.25)	−0.139	(−0.29, 0.04)
** NEU Avg**	−0.49*	(−0.63, −0.28)	−0.34	(−0.46, −0.12)	−0.139	(−0.28, 0.04)
** NEG Avg**	−0.47*	(−0.62, −0.26)	−0.35	(−0.47, −0.16)	−0.127	(−0.27, 0.04)
** POS Avg**	−0.49*	(−0.63, −0.28)	−0.41*	(−0.57, −0.20)	−0.182	(−0.33, 0.01)
** Averall Avg**	−0.51•	(−0.64, −0.31)	−0.39	(−0.51, −0.19)	−0.159	(−0.30, 0.02)

•p < 0.001. *p < 0.01.
