# Supplementary material for: Personality and Augmenting/Reducing (A/R) in auditory event-related potentials (ERPs) during emotional visual stimulation
Source: Sci Rep. 2017 Feb 6;7:41588. doi: 10.1038/srep41588 (PMC5292688; doi:10.1038/srep41588)
Supplement: Supplementary Information [file srep41588-s1.pdf]

Supplementary Information for

Personality and Augmenting/Reducing (A/R) in auditory event-related potentials (ERPs) during emotional visual stimulation

by Vilfredo De Pascalis, Francesca Fracasso, & Philip J. Corr

-- S1 –

-- Supplementary Table S1 --

Table S1. Statistics and zero-order correlations of valence (val) and arousal (arous) ratings for neutral (neu), negative (neg), positive (pos) pictures with BIS and FFFS measured with the RST-PQ, and SS measured with the ZKAPQ

Variabile	N	Mean	Std	Sum	Min	Max
BIS	39	54.79	11.85	2137	33	81
FFFS	39	26.24	5.29	1023	15	38
ZKAPQ-SS	39	98.75	11.21	3851	69	134
neg_val	39	2.44	0.49	95.08	1.91	3.38
pos_val	39	6.92	0.37	269.69	6.17	7.70
neu_val	39	5.29	0.45	206.31	4.30	5.97
neg_arous	39	6.20	0.77	241.63	4	7.59
pos_arous	39	6.12	0.76	238.54	4.86	7.77
neu_arous	39	4.21	0.70	164.15	2.43	5.60

Pearson Correlation Coefficients, N = 39<br>Prob > |r| with H0: Rho=0						
	neg_val	pos_val	neu_val	neg_arous	pos_arous	neu_arous
BIS	-0.164<br>*0.319*	0.150<br>*0.361*	-0.508<br>*0.001*	-0.200<br>*0.222*	-0.277<br>*0.088*	0.081<br>*0.623*
FFFS	-0.112<br>*0.498*	0.067<br>*0.687*	-0.222<br>*0.174*	-0.038<br>*0.818*	-0.314<br>*0.052*	0.021<br>*0.901*
ZKAPQ-SS	-0.474<br>*0.002*	0.075<br>*0.651*	-0.379<br>*0.017*	-0.149<br>*0.366*	0.035<br>*0.831*	0.200<br>*0.223*

-- S2 --

Results for RST traits and LORETA source localization. Since we have found that N1/P2 complex is sensitive to individual differences in RST traits, we have also tested these differences on standardized sLORETA scores to find regional cortical localizations of significant difference on current density within the N1 and P2 time windows.

In terms of individual differences in BIS, we found that high BIS, compared to low BIS, had a significantly lower activity at 101 ms (i.e., a maximal positive t value in the time window of N1 wave) in the sublobar insula, fusiform and parahippocampal gyri (BA13, BA31, BA23) of the left hemisphere (Supplementary Fig. S1-(a) and Supplementary Table S2). Interestingly, at 226 ms from

auditory stimulus onset (P2 wave) high BIS had lower activity than low BIS participants in the left superior parietal lobule (BA7), a higher activity in the right orbital frontal cortex (BA11, OFC), and a reduced activity in the right inferior parietal lobule (BA40; Supplementary Fig. S1-(b) and Supplementary Table S2).

For the FFFS we found that high FFFS, compared to low FFFS, had a significantly lower activity at 226 ms (P2 wave) in the right posterior cingulate (BA23 and BA30) and in the left postcentral gyrus in the parietal lobe (BA7; Supplementary Fig. S2-(a) and Supplementary Table S2).

-- Supplementary Fig. S1 --

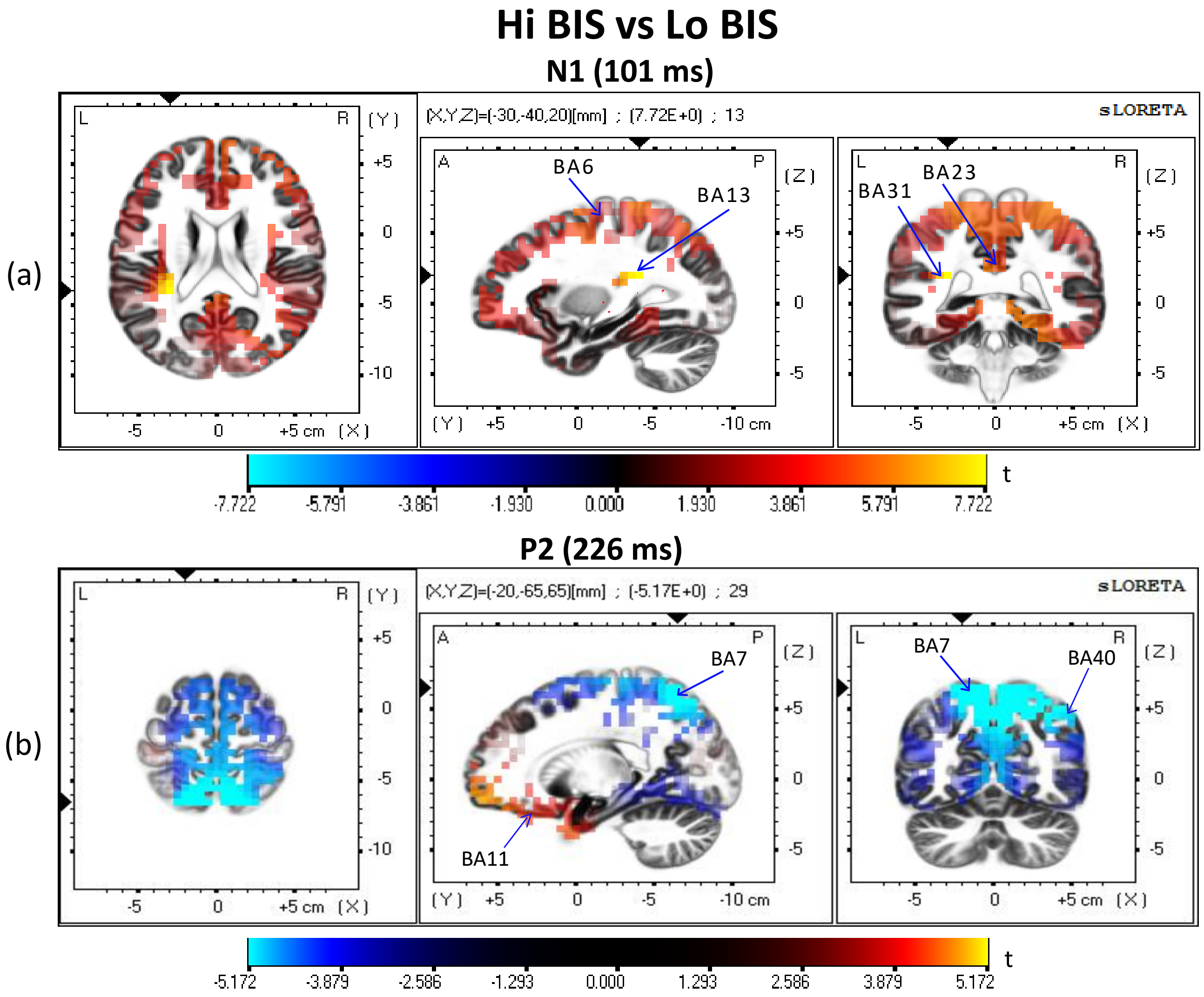

Supplementary Fig. S1. Panel (a): statistical parametric maps of sLORETA differences comparing high vs. low BIS groups (N1 wave). Note that a lower current density difference (yellow color) occurred in high BIS in the left sub-lobar insula, limbic parahippocampal gyrus, and right temporal fusiform gyrus (BA13, BA31, BA23, and BA6) at a time-frame of 101 ms (corrected threshold, $p < .01$). Panel (b): sLORETA difference maps comparing high vs. low BIS groups at 226 ms (P2 wave). A lower current density difference (light blue color) occurred in high BIS in the superior and inferior parietal lobule (BA7 and BA40), while a higher activity (yellow color) occurred in high BIS participants in the orbitofrontal gyrus (BA11).

-- Supplementary Fig. S2 --

Hi FFFS vs Lo FFFS : P2 (226 ms)

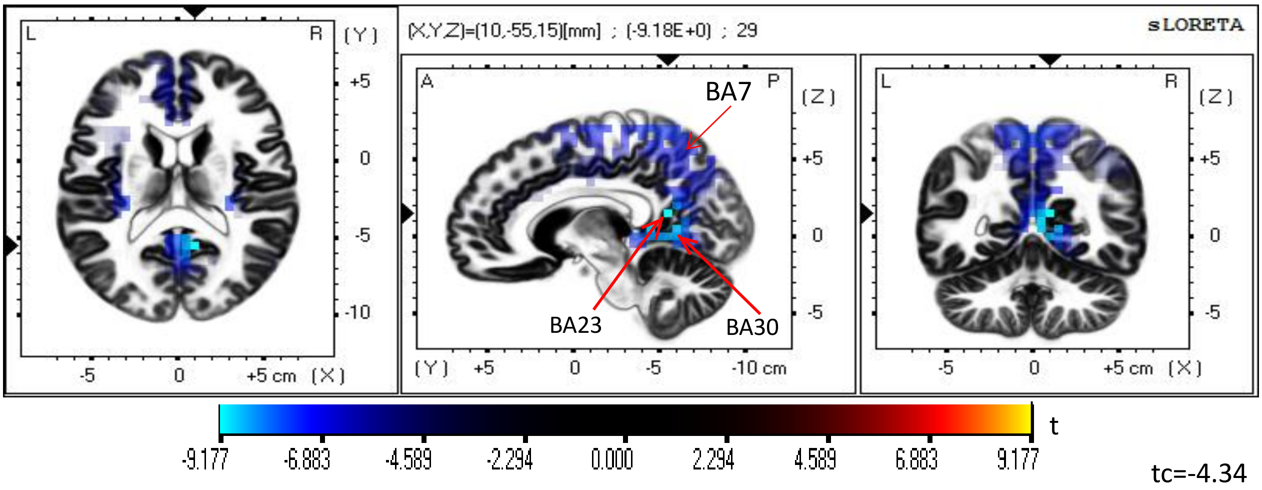

Supplementary Fig. S2. Statistical parametric maps of sLORETA differences comparing high vs. low FFFS groups (P2 wave). Note that a lower current density difference (light blue color) occurred in high FFFS in the posterior cingulate and postcentral gyrus (BA23, BA30, and BA7) at a time-frame of 226 ms (corrected threshold, $p < .01$).

--Supplementary Table S2--

Supplementary Table S2. MNI coordinates and Brodmann areas (BA) of significant differences in current density of N1 and P2 ERP waves between high BIS (N=19) vs Low BIS (N=19), high FFFS (N=15) vs Low FFFS (N=18). ERPs were elicited by auditory tones delivered using the augmenting/reducing paradigm.

ERP	X(MNI)	Y(MNI)	Z(MNI)	t(max)	BA	Lobe	Structure	No. of Voxels
Hi vs Lo BIS N1 (101 ms)	-30	-40	20	7.72	BA13	Sub-lobar	Insula	7
	-20	-45	25	7.07	BA31	Temporal	Fusiform Gyrus	12
	-5	-25	30	6.76	BA23	Limbic	Parahippocampal Gyrus	19
	30	0	65	6.46	BA6	Temporal	Fusiform Gyrus	10
Hi vs Lo BIS P2 (226 ms)	-20	-65	65	-5.17	BA7	Parietal	Superior Parietal Lobule	70
	15	45	-25	5.03	BA11	Frontal	Orbital Gyrus	16
	55	-55	45	-4.97	BA40	Parietal	Inferior Parietal Lobule	9
Hi vs Lo FFFS P2 (226 ms)	10	-55	15	-9.18	BA23	Limbic	Posterior Cingulate	10
	5	-55	5	-8.89	BA30	Limbic	https://1drv.ms/w/s!Anlw5bM8Uo0-inrJhu3emx_gWU51	28
	-5	-55	70	-7.63	BA7	Parietal	Postcentral Gyrus	54

Note: For the N1 wave, a positive value of t indicates that high (Hi) show lower current density than low (Lo) BIS (viceversa for a negative t value). For the P2 wave, a positive value of t indicates a higher current density in Hi than Lo BIS, or FFFS participants (viceversa for a negative t value). A negative X(MNI) coordinate indicates the left and a positive value the right hemisphere.

Discussion of LORETA findings. (see Supplementary Information online, section S4 and Supplementary Figs. S1, S2 and Table 4 online).

Finally, using sLORETA source localization method we found some overlapping significant differences in current densities within the N1 and P2 time windows between high and low BIS and FFFS levels. In particular, we found in high BIS, as compared to low BIS participants, a significant lower activity at 101 ms (N1 wave) in the sublobar insula, fusiform and posterior parahippocampal gyrus (BA13, BA31, BA23) of the left hemisphere and in the right fusiform gyrus (BA6) of the temporal lobe (Supplementary Fig. S1-(a) and Supplementary Table 4). In addition, high BIS had a lower activity at 226 ms (P2 wave) in the left superior parietal lobule (BA7), in the right inferior parietal lobule (BA40), and a higher activity in the right frontal orbital gyrus (BA11; Supplementary Fig. S1-(b) and Supplementary Table 4). High FFFS participants, compared to low ones, also disclosed lower activations at 226 ms from auditory stimulus onset, in the posterior cingulate and postcentral gyrus (BA23, BA30, and BA7; Supplementary Fig. S2-(a) and Supplementary Table 4). On this basis, it is reasonable to conclude that the reduced activity we observed in the above mentioned structures reflects the reduced sensitivity (or enhanced avoidance) to intense stimuli. This reduced cortical activity, observed in high BIS and FFFS individuals is consistent with the RST[1] that predicts, enhanced avoidance to intense/negative stimulations in high BIS and FFFS individuals. In line with this, high levels of stress have been associated with a reduction of both prefrontal cortex (PFC) and rostral anterior cingulate cortex (ACC) activity[2] and with our prior observation using auditory startle stimuli that lower self-report fear was associated with larger P200 amplitude, and enhanced current density in the medial and superior frontal gyrus (BA6)[3]. The present observations are also consistent with previous fMRI findings in a study by Bishop, et al. [4] designed to evaluate the role of anxiety in the processing of threat-related distractors. These authors found that anxiety is associated with reduced activity in the rostral ACC and lateral PFC indicating a loss in top-down control over threat-related distractors. Interesting, we also found a higher activity in the right OFC in high BIS that low BIS participants (Supplementary

Fig. S1-(b) and Supplementary Table 4). We think that the activation of OFC reflects the activation of neurophysiologic processes geared to enhanced cognitive control in these individuals[5].

Although LORETA provides good localization accuracy, a major limitation of the present study is in the fact that only 30 electrodes were used for source analysis. This reduces the spatial resolution, and with impaired spatial resolution, there is a smaller chance that LORETA will be able to separate two closely spaced sources. Thus, a greater number of recording electrodes is recommended in future studies to enhance spatial resolution. In conclusion, the present study served to test RST constructs of BIS, FFFS in terms of emotional modulation of auditory A/R and source localization of the ERP components, and indicated that these temperamental traits involve distinctive neurophysiological systems.

-- S4 --

Procedure for the Selection of Emotional Pictures

In a pilot study, a total of 30 female (22-36 age range, M = 24.6, SD = 2.6 yrs) Psychology students rated each image on valence and arousal to verify the initial selection. The ratings for positive, negative and neutral valence were obtained using a 9-point Likert Scale, ranging from 1 to 9 that varies from negative to positive with a neutral point (five). A similar scale was used to rate arousal levels, ranging from 1 (calm) to 9 (aroused). We selected from the IAPS all the positive pictures that had a score equal or higher than 7 in valence and 7 in arousal. We followed the same procedure for the negative images. We took the neutral images in a small interval around the 5-value for the valence (4.5 to 5.5). Since neutral images are typically rated lower in arousal relative to positive or negative images, this was quite difficult to select, from IAPS, a set of neutral images with a high arousal level such as that of emotional images. To remedy this problem, we selected from the IAPS the neutral pictures with the highest score on arousal, but we had to add 18 neutral surrealistic, potentially high arousable pictures downloaded from the web, as suggested in a previous study by Mourao-Miranda, et al. [6]. The final picture set consisted of 45 positive/high-arousal images, 45 negative/high-arousal images, and 46 neutral images. Below here are reported IAPS images used for each affective category:

Pleasant/high Arousal Stimuli:
1920, 2057, 2070, 2080, 2165, 2311, 2352, 4002, 4004, 4210, 4220, 4232, 4300, 4310, 4470, 4520, 4550, 4572, 4599, 4608, 4611, 4651, 4652, 4658, 4660, 4669, 4670, 4672, 4680, 4681, 4800, 5629, 8030, 8080, 8090, 8160, 8185, 8190, 8300, 8420, 8490, 8500, 8501, 8502, 8503.
Unpleasant/high Arousal Stimuli:
1300, 1321, 2710, 3015, 3030, 3051, 3060, 3140, 3160, 3170, 3181, 3250, 3350, 3530, 6260, 6300, 6312, 6313, 6350, 6370, 6510, 6530, 6540, 6550, 6560, 6571, 6821, 6830, 7380, 9005, 9006, 9180, 9181, 9252, 9300, 9320, 9340, 9405, 9410, 9433, 9800, 9810, 9910, 9911, 9920
Neutral/high Arousal Stimuli:
1616, 2214, 2381, 2485, 2840, 2880, 5220, 5410, 5470, 5535, 5720, 5740, 5750, 5789, 5920, 7205, 7207, 7230, 7270, 7350, 7352, 7495, 7496, 7502, 7510, 7640, 7820, 7830.
Eighteen high-arousal neutral stimuli were selected from the web. They are available on request at: vilfredo.depascalis@uniroma1.it

LORETA method

ERP responses were further analyzed using the last LORETA software version provided by the KEY Institute for Brain-Mind Research (University Hospital of Psychiatry, Zurich, Switzerland; http://www.uzh.ch/keyinst/NewLORETA/LORETA01.htm, version 25-04-2015).
sLORETA is a method for estimating the localization of cortical generators at specific time windows by providing a solution to the inverse problem by assuming that neighboring grid points are more likely to be synchronized than grid points that are far from each other and to find the best solution that is consistent with the scalp distribution [7]. sLORETA is mainly used to compute statistical maps from EEG data that indicate the locations of the putative underlying source generators[8]. These maps are derived by performing a location-wise inverse weighting of the results of a minimum norm least squares analysis with their estimated variances. This tool performs source localization in 6239 cortical gray matter voxels sized 5 mm^3 rather than 7 mm^3 offered by the previous LORETA version, and localization inference is based on standardized values of the current density estimates [9]. The solution space of LORETA is restricted to cortical and some hippocampal and amygdala gray matter defined via a reference brain from the Brain Imaging Center at the Montreal Neurological Institute[10,11] (MNI). The sLORETA implementation incorporates a 3-D shell spherical head model registered to a recognized anatomical brain atlas [12]. Individual 3-D electrodes are positioned by the Talairach coordinate system according to the spatial association between anatomical brain landmarks and scalp positions [13]. sLORETA enables the computation of statistical maps from ERP components data that indicate the locations of the underlying source processes with low error [8] and does not require a priori hypotheses regarding field distribution of active sources. In the present experiment the coordinates of the 30 electrode positions were applied to a probabilistic anatomical template of the Talairach Atlas (McConnell Brain Imaging Centre, Montréal Neurological Institute, McGill University). These Talairach coordinates were used to compute the LORETA transformation matrix and then to transform ERPs of each subject into sLORETA forms. This resulted in the corresponding 3D cortical distribution of the electrical neuronal generators for each subject. For source reconstruction, subtractions of ERP traces between high and low personality traits were assessed, for the N1 and P2 waves, respectively within time intervals of 100–140 ms and 200–225 ms. Statistical significance was assessed using a non-parametric randomization test [14]. sLORETA maps of high vs low personality levels were compared using a t test for independent samples with the aforementioned nonparametric permutation test. It is important to note that this localization is not a complete listing of all significantly different cortical areas, but a listing of the local maxima of these differences. Although simulations have shown that LORETA localization performed better than some other localization methods, LORETA, as with all ERP localization algorithms, has accuracy limitations [15]. Consequently, the significant brain regional differences reported in the present study between high and low levels of personality traits were examined only when N1/P2 differences between those traits were seen statistically significant.

References

1 Gray, J. & McNaughton, N. The Neuropsychology of Anxiety: an Enquiry into the Functions of the Septo-hippocampal System (2nd edn, Oxford: Oxford University Press). (2000).

2 Shin, L. M. *et al.* An fMRI study of anterior cingulate function in posttraumatic stress disorder. *Biological psychiatry* **50**, 932-942 (2001).

3 De Pascalis, V., Cozzuto, G. & Russo, E. Effects of personality trait emotionality on acoustic startle response and prepulse inhibition including N100 and P200 event-related potential. *Clinical Neurophysiology* **124**, 292-305 (2013).

4 Bishop, S., Duncan, J., Brett, M. & Lawrence, A. D. Prefrontal cortical function and anxiety: controlling attention to threat-related stimuli. *Nature neuroscience* **7**, 184-188 (2004).
5 Botvinick, M. M., Braver, T. S., Barch, D. M., Carter, C. S. & Cohen, J. D. Conflict monitoring and cognitive control. *Psychological review* **108**, 624 (2001).
6 Mourao-Miranda, J. *et al.* Contributions of stimulus valence and arousal to visual activation during emotional perception. *NeuroImage* **20**, 1955-1963 (2003).
7 Pascual-Marqui, R. D., Michel, C. M. & Lehmann, D. Low resolution electromagnetic tomography: a new method for localizing electrical activity in the brain. *International Journal of psychophysiology* **18**, 49-65 (1994).
8 Pascual-Marqui, R. D. Standardized low-resolution brain electromagnetic tomography (sLORETA): technical details. *Methods and findings in experimental and clinical pharmacology* **24**, 5-12 (2002).
9 Wagner, M., Fuchs, M. & Kastner, J. Evaluation of sLORETA in the presence of noise and multiple sources. *Brain topography* **16**, 277-280 (2004).
10 Mazziotta, J. *et al.* A probabilistic atlas and reference system for the human brain: International Consortium for Brain Mapping (ICBM). *Philosophical transactions of the Royal Society of London. Series B, Biological sciences* **356**, 1293-1322, doi:10.1098/rstb.2001.0915 (2001).
11 Collins, D. L., Neelin, P., Peters, T. M. & Evans, A. C. Automatic 3D intersubject registration of MR volumetric data in standardized Talairach space. *Journal of computer assisted tomography* **18**, 192-205 (1994).
12 Talairach, J. & Tournoux, P. *Co-planar stereotaxic atlas of the human brain*. (Thieme, 1988).
13 Towle, V. L. *et al.* The spatial location of EEG electrodes: locating the best-fitting sphere relative to cortical anatomy. *Electroencephalography and clinical neurophysiology* **86**, 1-6 (1993).
14 Nichols, T. E. & Holmes, A. P. Nonparametric permutation tests for functional neuroimaging: a primer with examples. *Human brain mapping* **15**, 1-25 (2002).
15 Pascual-Marqui, R. D. Review of methods for solving the EEG inverse problem. *International journal of bioelectromagnetism* **1**, 75-86 (1999).
